# Tumor-Educated Platelets: A Review of Current and Potential Applications in Solid Tumors

**DOI:** 10.7759/cureus.19189

**Published:** 2021-11-01

**Authors:** Joyce Varkey, Theodore Nicolaides

**Affiliations:** 1 Pediatric Hematology Oncology, New York University Langone, New York, USA

**Keywords:** liquid biopsy, solid tumor, tumor-educated platelet, lung cancer, oncology

## Abstract

In this current era of precision medicine, liquid biopsy poses a unique opportunity for an easily accessible, comprehensive molecular profile that would allow for the identification of therapeutic targets and sequential monitoring. Solid tumors are definitively diagnosed by analyzing primary tumor tissue, but surgical sampling is not always sufficient to generate a comprehensive genetic fingerprint at the time of diagnosis, or an appropriate means for continued monitoring. Platelets are known to have a dynamic, bidirectional relationship with tumors, acting beyond their role of hemostasis. Tumor-educated platelets (TEP) are modified by the tumor in multiple ways and act as a carrier and protector of metastasis. Data so far have shown that the mRNA in TEP can be harnessed for cancer diagnostics, with many potential applications.

## Introduction and background

Liquid biopsy is a promising tool and potentially revolutionary technique in the realm of cancer diagnostics. Currently, solid tumors are definitively diagnosed by pathologic analysis of tumor tissue, except in very rare cases. However, surgery alone does not always provide enough tissue to create a full genetic profile for appropriate assignment to tailored therapy [1,2]. Liquid biopsy overcomes the problem of accessibility to the tumor tissue and is much less invasive, which allows for more frequent monitoring of tumor dynamics. It potentially allows for a more comprehensive molecular profile as it is more reflective of clonal heterogeneity. In principle, liquid biopsy should reflect all subclones present at that specific time point; therefore, it should reflect changes in the clonal populations over time via selective pressures, as well as the biochemical changes acquired during blood-borne dissemination, which are not present in the primary tumor [3]. The era of precision cancer medicine has heightened the need for high-quality diagnostic material. Hence, liquid biopsy represents a potential complement to surgical diagnosis in the current landscape and may eventually become an alternative.

Existing liquid biopsy assays

The liquid biome, which consists of cerebrospinal fluid, saliva, serum, plasma, seminal plasma, and urine, has been investigated as a potential source for liquid biopsy. In particular, blood-based biosources for liquid biopsy include the mononuclear cell fraction (which encompasses leukocytes, circulating tumor cells, circulating endothelial cells), plasma and serum (which encompasses extracellular vesicles, cell-free DNA [cfDNA], cell-free RNA, plasma proteins, and metabolites), and tumor-educated platelets (TEP) [4,5].

Although the first commercially available liquid biopsy assay was introduced in 2000, it was not until 2016 that the Food and Drug Administration (FDA) first approved liquid biopsy tests. The first was the cobas® epidermal growth factor receptor (EGFR) mutation test from Roche Molecular Diagnostics which uses cfDNA in blood to detect the *EGFR* gene mutation to guide clinical decision-making in lung cancer [6]. Tyrosine kinase inhibitors (TKIs) that target the *EGFR* have improved progression-free survival in patients with *EGFR* mutations compared to platinum-doublet chemotherapy. However, the *EGFR T790* point mutation is the most common mechanism of acquired resistance to first- and second-generation EGFR-TKIs (gefitinib, erlotinib, afatinib). If the *T790* mutation is detected by the cobas test, the clinician can choose to switch to osimertinib, which is a third-generation EGFR-TKI that bypasses the resistant mutation. The same year, the Epi proColon test from Epigenomics AG was also approved; this test identifies the methylation status of the *SEPT9* promoter in plasma cfDNA. Although it has a low sensitivity for early-stage I colorectal cancer (CRC), hypermethylation of the *SEPT9* promoter has been positively associated with CRC [7]. In 2020, two liquid biopsy assays that utilize next-generation sequencing were approved. Guardant360 CDx has an FDA-approved panel of 55 genes, while the FoundationOne CDx has 324 genes. Using cfDNA, they function as a companion diagnostic test for the following therapies: rucaparib (identifying *BRCA1* and *BRCA2* genes in patients with ovarian cancer), alectinib (anaplastic lymphoma kinase [ALK] rearrangements in non-small-cell lung cancer [NSCLC]), alpelisib (*PIK3CA* gene mutations in breast cancer), olaparib (mutations in *BRCA1* and 2 and *ATM* genes in metastatic castration-resistant prostate cancer), and vitrakvi (*NTRK* fusions, location-agnostic). Liquid biopsies can also determine the microsatellite instability (MSI-H) status and the tumor mutational burden (TMB) [8].

Platelets and cancer: an intersection

Platelets assist in thrombosis and hemostasis, conduct immune surveillance and communication, aid vessel remodeling, and abet inflammation [9-11]. They also play an active role in cancer biology, and, in turn, are dynamically affected by cancer, thus providing opportunities to use TEPs and their RNA repertoire for diagnosis, prognosis, prediction, and surveillance [10]. The interaction between platelets and cancer was first noted by Trousseau in 1868 when he noted that spontaneous coagulation is common in patients with cancer. A decade later, Billroth described clots containing malignant cells and supported the hypothesis that thrombi were an agent of metastasis. Platelet morphology was described in the early 1900s, but it was not until 1966 that platelets were found to be capable of protein synthesis despite being anucleate. Since 2010, many discoveries have been made in rapid succession that continue to fortify the bidirectional link between platelets and cancer [12,13].

**Figure 1:**
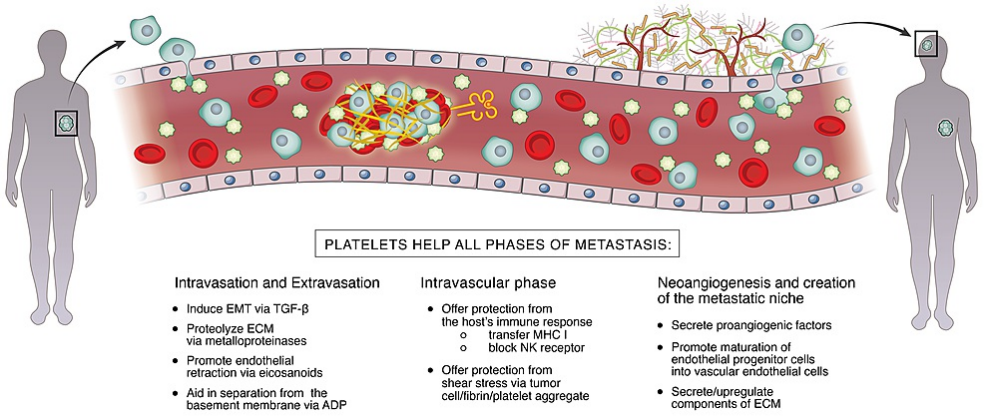
The intersection between platelets and cancer. EMT = endothelial mesenchymal transition; TGF-β = transforming growth factor-beta; ECM = extracellular matrix; ADP = adenosine diphosphate; MHC = major histocompatibility complex; NK = natural killer cell

Originally, it was postulated that mechanical embolization was the process by which activated and aggregated platelets contribute to metastasis [14]. However, it is now clear that the interaction between platelets and cancer is far more complex. Systemic spread of cancer consists of a carefully orchestrated series of events, with an intravascular phase sandwiched by intravasation into the vessel and extravasation into the niche [10,15]. Platelets also contribute to the formation of new blood vessels and the metastatic niche. This process is illustrated in Figure 1.

Platelets promote invasion of the tumor into the local vasculature by first adhering the tumor cell to the endothelium of the vessel and then disrupting the extracellular matrix. It does so by secreting growth factors, such as transforming growth factor-beta (TGF-β), which induces the epithelial-mesenchymal transition. TGF-β also aids in the adherence of the tumor cell. The extracellular matrix is proteolyzed by matrix metalloproteinases secreted by platelets and/or tumor cells. Platelets release eicosanoid metabolites such as thromboxane to prompt endothelial cell retraction, creating apertures through which the tumor cells intravasate. Adenosine diphosphate (ADP), which is also released by activated platelets, plays an important role in the process by increasing the permeability of the basement membrane [11].

During the intravascular phase, platelets aid and abet circulating tumor cells (CTCs) by providing protection from shear stress, as well as evasion of the host’s immune response by way of multiple mechanisms. Via surface molecules such as tissue factor and P-selectin ligands, CTCs adhere to platelets, which protect them physically from the shear forces of the vessels [16]. GPIIb/IIIa integrins on the platelet also interact with αvβ integrins of the tumor cells to protect the tumor from these shear forces [11]. Platelets provide protection against natural killer (NK) cell lysis by forming a cell-fibrin-platelet aggregate [16], binding its receptors, and transferring platelet-derived major histocompatibility complex (MHC)-1 [5,16]. Activated platelets release express glucocorticoid-induced tumor necrosis factor (TNF)-related ligand which binds to the NK cell and blocks the secretion of interferon-gamma (which activates NK cells) [9]. Further, platelet- and CTC-derived TGF-β limits CD4+ and CD8+ T-cell functions as well [16].

Once the metastatic cell finds its micrometastatic niche, it needs to exit the blood vessel. All the mechanisms that the platelets employed to help with intravasation are utilized for extravasation.

Platelets secrete CXCL5 and CXCL7, chemokines that mediate the recruitment of host-derived cells that build tumor stroma. It has also been shown that platelet ADP receptor P2Y1 upregulates lung fibronectin, which is a major component of the extracellular matrix [16].

Neoangiogenesis is critical for any cancer, but particularly that are larger than 2 mm [16]. Platelets play a critical role throughout the entire process but must be activated to contribute. Tumor cell-induced platelet activation (TCIPA) is prompted by either direct contact between tumor and platelet, or when the tumor interacts with the agonistic mediators released by platelets, and is characterized by platelet aggregation, adhesion, and an increase in both platelet numbers and platelet-derived angiogenic factors [5,11]. Platelets release both inhibitory and stimulatory factors; however, most models show that the pro-angiogenic effects predominate [15]. The most important pro-angiogenic substances include, but are not limited to, vascular endothelial growth factor (VEGF), platelet-derived growth factor (PDGF), transforming growth factor (TGF), insulin-like growth factor (ILGF), sphingosine-1-phosphate, basic fibroblast growth factor (bFGF), endothelial cell growth factor (ECGF), epidermal growth factor, and angiopoietin-1 (ANGPT-1) [5,15,16]. VEGF may play the most important role in this process and has been shown to predict cancer progression [17]. Additionally, platelets recruit and promote the maturation of endothelial progenitor cells (EPCs) from the bone marrow into endothelial cells of the neovasculature [16].

Education of platelets

**Figure 2:**
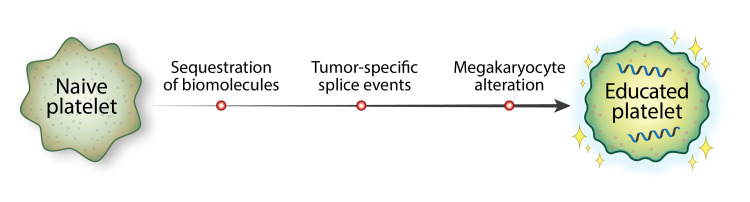
The platelet education process.

In the process of interacting with tumor cells, platelets become “educated” and result in an altered RNA profile. This education is completed through three primary avenues: sequestration of tumor-specific biomolecules, tumor-specific splice events, and megakaryocyte alteration (Figure 2) [5,12,13,18,19].

Platelets continuously exchange circulating nucleic acids and proteins via vesicle-mediated transport mechanisms with the tumor and its microenvironment, including immune cells, endothelial cells, and stromal cells. The platelets then harbor and express these tumor-specific elements [5]. Some transferred biomolecules include tumor-specific transcripts such as EGFRvIII, echinoderm microtubule-associated protein-like 4 (EML4)-ALK, Kirsten rat sarcoma viral oncogene homolog (KRAS), EGFR, PIK3CA variants, kallikrein-related peptidase (KLK)2, KLK3, and neuropeptide Y (NPY) [13].

Taking up tumor elements is a direct method of education. Indirectly, platelets are modified by post-transcriptional splicing. Although platelets are anucleate, they contain many types of RNA, such as precursor messenger RNA (pre-mRNA), messenger RNA (mRNA), ribosomal RNA (rRNA), small nuclear RNA (snRNA), small nucleolar RNA (snoRNA), transfer RNA (tRNA), microRNAs (miRNA), long noncoding RNAs (lncRNAs), circular RNAs (circRNA), antisense RNA (asRNA), and mitochondrial DNA, as well as a spliceosome and a ribosome. Specific splice events occur in reaction to stimuli such as platelet activation [5,10].

There is also evidence of external modification of platelets, both via tumor-bone marrow communication (by which a tumor may alter a megakaryocyte’s transcriptional profile via cytokines or extracellular vesicles [20]), or by affecting the platelet subpopulations [5]. Younger, reticulated platelets are more RNA-rich and in a more inflammatory, pro-active state; it has been shown that in patients with cancer, younger platelets are more common than older platelets, which may enrich for processes that are critical for tumor progression [5].

The technique of RNA sequencing

The thromboSeq technology, which generates a whole transcriptome sequence from negligible amounts of platelet RNA, was first developed in the Netherlands at the VU University Medical Center [5].

## Review

Tumor-educated platelets and adult solid tumors: a review of the literature

In 2015, Best et al. published a seminal article detailing their work creating distinct onco-signatures for six primary cancer types. RNA sequencing was performed on platelet samples isolated from 55 healthy individuals and 228 patients with cancer. A total of 1,453 out of 5,003 (29%) mRNAs were increased, and 793 out of the same 5,003 mRNAs (16%) were decreased in TEPs compared to platelet samples of healthy donors. Using leave-one-out cross-validation support vector machine (SVM) learning, it was possible to develop a predictive algorithm, or “signature,” that could distinguish between healthy donors and cancer patients with 96% accuracy, as well as differentiate between six primary tumor types (NSCLC, CRC, glioblastoma (GBM), pancreatic cancer, breast cancer, and hepatobiliary cancer) with 71% accuracy [19]. The following sections describe how TEPs have been harnessed for a wide spectrum of oncological conditions in adults.

Lung Cancer

Globally, lung cancer is the most commonly diagnosed malignancy (approximately 2.89 million new cases in 2020) and the leading cause of cancer-related death (1.74 million deaths in 2020), with an average five-year survival rate of 15%. Unfortunately, it is often diagnosed at a late stage, and approximately two-thirds of patients have metastatic disease at the time of diagnosis. The gold standard for diagnosis is tissue biopsy [21]. In advanced-stage NSCLC, therapies that target genetic alterations have been shown to be effective, especially for those with *EGFR* mutations, EGFR-TKIs, and for those with *EML4-ALK* mutations, an ALK inhibitor (such as crizotinib). For lung cancers, in particular, there are high failure rates for mutation evaluation (i.e., 32%, 27%, and 35% for EGFR, KRAS, and ALK tests, respectively) with image-guided percutaneous transthoracic core-needle biopsies [22].

In 2015, using TEP-derived RNA analysis, lung cancer was distinguished from other primary tumor types with an average accuracy of 77%. Subtypes of lung cancer were also identified: *EGFR* mutations were identified with 87% accuracy, *MET* amplification with 91% accuracy, and *KRAS* mutations with 90% accuracy [19]. In 2016, Nillson et al. showed that *EML4-ALK* mutations could be identified with 65% sensitivity. In one patient from that cohort, serial monitoring of the rearrangement via TEP analysis predicted resistance to crizotinib two months prior to radiographic progression [23]. In 2017, RNA biomarker panels were selected from platelet-sequencing libraries, allowing for the detection of early and late-stage NSCLC (n = 106 in early-stage cohort, 81% accuracy; n = 518 in late-stage cohort, 88% accuracy) [24].

A few other biomarkers have also been identified. Sheng et al. identified 48 biomarker genes that can be used to identify cancer with an accuracy of 89% [25]. Xue et al. found that expression of apoptotic chromatic condensation inducer 1 (ACIN1) mRNA was increased in patients with lung cancer, with a sensitivity of 72% [26]. Xing et al. discovered that NSCLC overexpressed Integrin α-IIb (ITGA2B) [27]. Li et al. identified 20 genes that are 78% sensitive for detecting adenocarcinoma in situ [28]. These may eventually be applied to clinical practice, especially to identify cancer earlier than is currently possible, or to assign tailored therapy appropriately.

Breast Cancer

Breast cancer is the most common cancer type in women globally, with the following three subtypes: estrogen receptor or progesterone receptor-positive (ER+/PR+), which are treated with endocrine therapy; human epidermal growth factor receptor-positive (HER2+), which is treated with trastuzumab; and triple-negative tumors, which do not have any of the receptors and are associated with a high mortality rate. Breast cancers demonstrate high intratumoral heterogeneity, which makes a comprehensive molecular fingerprint even more important [29]. Using TEP-derived RNA profiles, Best et al. were able to successfully identify breast cancer in patients with an average accuracy of 62%; they also identified three subtypes (HER2+, PIK3CA, and triple-negative) [19].

Glioblastoma

GBM is unfortunately both the most common and the most lethal primary brain tumor in adults. Average survival is 12-15 months after diagnosis, with a five-year survival of less than 5%. The current standard of care is radiotherapy and temozolomide after maximal resection. GBM is a heterogeneous entity - not only is it clinically variable but also demonstrates tremendous genomic and morphological variability [30-32]. Therefore, tailoring therapies to a patient’s particular molecular subtype of GBM is critical. However, traditional biopsies are invasive and repeated biopsies are impractical, if not impossible.

In 39 patients with GBM, 33 were accurately identified using a TEP signature. EGFRvIII, a gene rearrangement that is specific to GBM, was found in 80% of patients with GBM but not in healthy donors. Therefore, this genetic rearrangement was used in the onco-signature for GBM [19].

In 2020, data were expanded to investigate whether TEP signatures could be used to distinguish not only diseased from healthy patients but also cancer from inflammatory processes, as well as to see if cancer progression could be measured with the TEP signature [20]. To that end, platelets were collected and isolated from 89 patients with primary GBM on the day of first tumor resection. Blood was then collected at several following time points during the concurrent chemoradiation phase, together with magnetic resonance imaging (MRI) tumor visualization (52 unique patients, 151 platelet samples in total, two to nine samples per patient). Blood was also collected from 126 patients with one or multiple brain metastases. Brain metastases were diagnosed from different primary tumors (NSCLC = 85, breast = 15, melanoma = 15, renal cell cancer = 7, CRC = 1, esophagus cancer = 1, pancreatic cancer = 1, unknown = 1). Most of these patients had metastatic lesions to other organs as well. In total, 86 patients had clinically stable, relapsing-remitting multiple sclerosis (MS). Lastly, 353 asymptomatic healthy controls were recruited, without self-reported symptoms of neurological disorders or cancer. All four groups had a total series size of 805 samples. To minimize potential confounding factors, sample sets were matched for age and whole blood storage time, resulting in a matched series of 48 patients in each group: with GBM at the time of first resection, patients with brain metastasis, patients with MS, and healthy controls. For all these analyses, the total matched sample series were randomly separated into training, evaluation, and validation series.

The training samples were employed to select a spliced RNA biomarker panel and to build a machine learning SVM algorithm, whereas the evaluation series was employed to further optimize the spliced RNA biomarker panel and performance of the SVM algorithm by swarm intelligence. First, an algorithm biomarker panel of 212 platelet spliced RNAs was calculated to distinguish GBM versus brain metastasis and MS, with an average accuracy of 85%. For GBM versus brain metastasis, a biomarker panel of 333 platelet-spliced RNAs was selected, with an average accuracy of 87%. For GBM versus MS, a biomarker panel of 200 platelet-spliced RNAs was selected, with an average accuracy of 92%. For pre-resection GBM versus asymptomatic healthy controls, a panel of 200 markers was used, with an average accuracy of 96%.

Further, the algorithm can also provide, for each sample, a quantitative score ranging from 0-1, representing the classification confidence score, or a TEP score. The more pronounced the GBM signal in a certain sample, the more the sample has a classification confidence score toward 1. The TEP score of the samples collected just before tumor resection was significantly higher compared with those collected during the follow-up period (0.91 vs. 0.44 vs. 0.24).

To determine whether TEP-derived spliced RNA profiles mirror the disease burden in the longitudinal sample collection, the TEP score was analyzed in 52 patients who were treated for their GBM. When comparing preoperative baseline blood samples with samples collected after tumor resection (median number of days after resection: 19; range: 10-33 days), a mean decrease in TEP score was observed after resection of 0.40. However, an exact “half-life” of the tumor signal in TEPs could not be determined.

Subsequently, for each available time point, the TEP score was matched with the available MRI of the tumor. Interestingly, for some patients, the TEP score increased in the blood prior to radiological tumor progression, potentially indicating that a blood test may precede clinical and/or radiological tumor evolution. Although the correlation varied in individual cases, these results indicate that the TEP score could be employed for GBM therapy monitoring. Next, the digital swarm algorithm was used to select 267 RNAs to create a “progressors vs. nonprogressors” panel. The average accuracy was 83%. Importantly, the four patients with false-positive progression included in the validation series were classified correctly as nonprogressors.

Prostate Cancer

Though prostate cancer is among the most common cancers in men globally, the overall five-year survival rate is over 98%. This is a clinical scenario in which liquid biopsies would prove helpful during a “watchful waiting” period. However, a small subset has an aggressive disease that may become castration-resistant. There are a few targeted therapies, such as enzalutamide or abiraterone, that may be helpful to these patients who would otherwise have a poor prognosis. Tjon-Kon-Fat et al. found that KLK2, KLK3, and FOLH1 were associated with higher levels of serum prostate-specific antigen, and may serve as indirect markers of tumor burden. However, NPY and PCA3 did not have any measurable association [33].

Colorectal Cancer

CRC is another tumor with a high mortality rate; most patients are diagnosed in advanced stages because early stages are typically asymptomatic. Colonoscopy is the standard of preventative care but has several disadvantages including financial and resource costs, risk of complications, and patient discomfort. The amount of ctDNA and CTCs needed is too high to be feasible as a method of screening [34]. The pan-cancer test published in 2015 showed an average accuracy of 60% for distinguishing CRC from other tumor types using TEPs, which is a promising finding [19]. Yang et al. found that TIMP metallopeptidase inhibitor 1 (TIMP1) mRNA levels were higher in platelets from patients with CRC compared to those from healthy volunteers or patients with inflammatory bowel diseases, which may prove to be another helpful signature [34].

Pancreatic Cancer

Pancreatic cancer continues to be a devastating diagnosis; the five-year survival rate remains below 5%. The only blood-based biomarker is carbohydrate antigen 19-9 (CA 19-9), but it is neither sensitive nor specific enough to be useful as a diagnostic marker for small nonmetastatic lesions [35]. The pan-cancer test demonstrated 56% accuracy in identifying pancreatic cancer and could identify a subset with *KRAS* mutations [19].

Hepatobiliary Cancer

Hepatobiliary cancer is another cancer with a poor prognosis; the five-year survival rate is nearly 20% [36]. In 2015, Best et al. showed 56.5% accuracy in detecting hepatobiliary cancer. They were also able to identify a subset with *KRAS* mutations [19]. Within the three types of gastrointestinal adenocarcinomas, Best et al. could correctly classify CRC, pancreatic cancer, and hepatobiliary cancer (N = 90), with 76% overall accuracy. In 2020, Asghar et al. demonstrated that TGF-β, NF-κB, and VEGF transcripts were increased and AKT and PI3K transcripts were decreased in those with hepatocellular cancer compared to healthy controls. In particular, AKT and PI3K were useful for detecting early-stage hepatocellular carcinoma [36].

Nasopharyngeal Carcinoma

In 2019, Wang et al. described two TEP microRNAs (miR-34c-3p and miR-18a-5p) that were upregulated in nasopharyngeal carcinoma compared to microRNAs isolated from healthy donor platelets (p < 0.001). However, there was not a significant difference in the levels of microRNA among different TNM stages, and they did not compare the levels between patients with nasopharyngeal carcinoma and cervical node metastatic disease, which is what they are often misdiagnosed as [37].

Applications to pediatrics

Thus far, there is no published data on TEPs in relation to pediatric tumors. Though it stands to reason that RNA signatures of pediatric solid tumors can be isolated based on the data from adult tumors, the pathology of tumors found in children is vastly different. For instance, the most common primary brain tumor in adults is GBM, whereas in children the most common type is low-grade pilocytic astrocytoma [38].

There is, however, a plethora of data that supports other modalities as a means of liquid biopsy, such as cfDNA, circulating tumor DNA (ctDNA, a small fraction of cfDNA), tumor-derived exosomes, and metabolites [39]. Unfortunately, CTCs have not proven to be as powerful as a method as had been hoped because their quantities are often quite minute [40].

There have been several reviews published in recent years that discuss the potential applications of cfDNA and ctDNA in neuroblastoma, different types of renal malignancies, osteosarcoma, and rhabdomyosarcoma. These liquid biopsies quantify myriad biomarkers, but some may be useful for diagnosis, risk stratification, or monitoring therapy response [39,41-46]. For example, researchers have identified copy number alterations, myc-n translocations, or activating *ALK* mutations, which are negative prognostic markers for neuroblastoma [41,43]. Hallmark genetic fusions have been detected in Ewing sarcoma and alveolar rhabdomyosarcoma (EWSR1 and PAX3-FOXO1, respectively) [41,47,48]. Renal malignancies are especially difficult to diagnose because biopsy carries a high risk of tumor rupture. Therefore, non-Wilms tumors may be misdiagnosed. ctDNA has been used to detect internal tandem duplications in *BCL-6 co-repressor*, which is a hallmark of clear cell sarcoma of the kidney [49]. Several reviews have expanded upon liquid biopsy techniques under investigation for pediatric central nervous system tumors [38,40,41,45,50].

All of these tumors present issues with repeat biopsies, and physicians must often wrestle with the question of whether or not a mass seen on imaging is true progression, scar tissue, or inflammatory tissue, resorting to positron emission tomography scans, which are helpful but imperfect. Isolating a pre-resection TEP mRNA signature, in comparison to signatures obtained at regular intervals, provides an opportunity to better determine if a radiological finding is true progression.

Another possible application is for children with neurofibromatosis who present with tumors such as plexiform neurofibromas. As these are not often biopsied, correlating mRNA signatures from TEPs to existing databases for RNA sequencing data could allow for more precise monitoring of tumor progression, as well as transformation to a malignant peripheral nerve sheath tumor. The current standard is clinical and radiographic monitoring. If transformation were identified using a molecular fingerprint, it could allow the physician to target the lesion much sooner and potentially improve outcomes.

## Conclusions

Platelets, which are educated by way of multiple avenues, and function as a vector of metastasis, may be a wellspring of easily accessible, more comprehensive tumor molecular data. The detection of circulating tumor components is clinically relevant as possible biomarkers for diagnosis, subsequent monitoring for recurrence, and identification of therapeutic targets and resistance mechanisms. Thus far, data from many common adult tumors strongly suggest that TEP-derived mRNA onco-signatures may be a feasible practice. More data are needed to validate this in larger, more diverse populations including pediatric patients.
